# Vitamin D Attenuates Inflammation and Mitochondrial Dysfunction in Experimental Models Relevant to Connective Tissue Disease–Associated Pulmonary Arterial Hypertension

**DOI:** 10.1002/fsn3.71620

**Published:** 2026-03-12

**Authors:** Yansheng Jin, Chaoqi Lu, Yi Ling, Juan Chen, Xiaozhong Li

**Affiliations:** ^1^ Department of Nephrology and Immunology Suzhou Wuzhong People's Hospital Suzhou Jiangsu China; ^2^ Department of Central Laboratory Suzhou Wuzhong People's Hospital Suzhou Jiangsu China; ^3^ Pharmacy Department Suzhou Wuzhong People's Hospital Suzhou Jiangsu China; ^4^ Department of Ultrasound Yinshanhu Hospital Suzhou Jiangsu Province China; ^5^ Department of Nephrology and Immunology Children's Hospital of Soochow University Suzhou Jiangsu Province China

**Keywords:** inflammation, mitochondrial function, nutritional status, pulmonary arterial hypertension, vitamin D

## Abstract

Vitamin D (VD) deficiency is prevalent in chronic inflammatory disorders and has been implicated in cardiopulmonary diseases. This study investigated whether VD, as a nutritional factor, modulates inflammatory and mitochondrial homeostasis in experimental pulmonary arterial hypertension (PAH) models and explored mechanisms with potential relevance to connective tissue disease–associated PAH (CTD‐PAH). A monocrotaline‐induced rat model and PDGF‐BB/hypoxia–treated pulmonary artery smooth muscle cells (PASMCs) were used. Hemodynamics, right ventricular remodeling, and vascular structure were assessed by catheterization and histology. Inflammatory cytokines, mitochondrial function, and apoptosis were evaluated by Enzyme‐Linked Immunosorbent Assay (ELISA), JC‐1, ROS, ATP assays, and related protein analyses. Western blot, quantitative real‐time polymerase chain reaction (qRT‐PCR), PARP1 activity, and co‐immunoprecipitation were performed to examine NF‐κB regulation via the Hes1‐PARP1 axis and TNFAIP3. VD supplementation improved pulmonary hemodynamics, reduced right ventricular hypertrophy, and attenuated pulmonary vascular remodeling. In PASMCs, VD suppressed abnormal proliferation, promoted apoptosis, and restored mitochondrial homeostasis. Mechanistically, VD downregulated the Hes1‐PARP1 axis while upregulating TNFAIP3, leading to inhibition of NF‐κB activation and inflammatory signaling. VD modulates inflammatory and mitochondrial homeostasis in experimental PAH models through coordinated regulation of the Hes1‐PARP1 axis and TNFAIP3. These findings support VD as a nutritional factor involved in modulating inflammatory and mitochondrial homeostasis during early PAH progression, and provide mechanistic support for VD‐related nutritional strategies with relevance to CTD‐PAH–associated pulmonary vascular remodeling.

## Graphical abstract

Mechanism study of VD mediated inflammatory and mitochondrial homeostasis in early PAH progression.

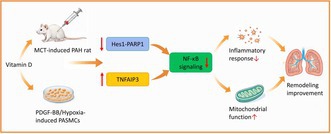

## 1 Introduction

Nutritional status is increasingly recognized as an important determinant of susceptibility to, and progression of, chronic cardiovascular diseases because nutrient availability influences immune regulation and cellular energy metabolism (DeBerge et al. 2023; Thorp and Karlstaedt 2024). Micronutrient deficiencies have been linked to impaired mitochondrial function and disrupted redox balance, processes implicated in chronic cardiovascular disorders (Qiu et al. 2025). Pulmonary vascular disease is an integral subset of cardiovascular disease in which inflammatory and metabolic remodeling drive pulmonary vascular remodeling (Steinhauser and Maron 2024). Increased pulmonary vascular resistance elevates right ventricular afterload, promoting right ventricular remodeling and ultimately right heart failure (Neelakantan et al. 2024). Therefore, defining nutrition‐related mechanisms linking inflammation and mitochondrial dysfunction to pulmonary vascular remodeling may inform nutritional strategies for cardiopulmonary risk reduction.

Among micronutrients, vitamin D (VD) is distinctive due to its dual identity as a dietary‐derived nutrient and a hormone‐like regulator that integrates environmental exposure, diet, and host physiology (Carlberg et al. 2023; Giustina et al. 2024). Importantly, VD insufficiency/deficiency is highly prevalent worldwide. A large global synthesis covering 2000–2022 estimated that the prevalence of serum 25‐hydroxyvitamin D (25(OH)D) concentrations < 30 nmol/L was approximately 15.7% across populations aged ≥ 1 year, underscoring that inadequate VD status represents a substantial public‐health burden (Cui et al. 2023). This high background prevalence is of public‐health and clinical relevance because low VD status has been associated with altered immune regulation and inflammatory responses, and VD signaling has been implicated in mitochondrial function and cellular redox homeostasis—processes central to chronic cardiopulmonary disease progression (Artusa and White 2025; Zhang et al. 2024).

Pulmonary arterial hypertension (PAH), particularly connective tissue disease–associated PAH (CTD‐PAH), is a severe cardiopulmonary complication characterized by progressive pulmonary vascular remodeling and eventual right ventricular failure, and effective early‐stage interventions remain limited (Rodolfi et al. 2024). Accumulating evidence supports a link between VD status and pulmonary hypertension (Atamañuk et al. 2019). Observational studies report that VD deficiency is frequently present in PAH and correlates with worse clinical profiles (Adao et al. 2024; Callejo et al. 2024). In addition, genetic‐inference evidence strengthens this association: a two‐sample Mendelian randomization study reported an inverse relationship between circulating 25(OH)D levels and pulmonary hypertension risk, supporting a potential causal contribution of low VD status (Chao et al. 2024). Experimental studies further indicate that VD deficiency may promote pulmonary vascular remodeling, whereas VD supplementation improves pulmonary vascular architecture and right ventricular function (Callejo et al. 2021; Shah et al. 2023). However, the nutrition‐sensitive molecular mechanisms linking VD status to key pulmonary vascular pathological processes relevant to CTD‐PAH remain insufficiently defined.

Inflammation and mitochondrial dysfunction are closely interconnected and have been implicated as key drivers of PAH progression (Ryanto et al. 2023; Si et al. 2025). In pulmonary vascular cells, persistent activation of inflammatory transcriptional programs—particularly NF‐κB—promotes cytokine production and vascular remodeling, whereas mitochondrial dysfunction (altered membrane potential, reactive oxygen species generation, disturbed calcium handling, and reduced ATP production) contributes to the hyperproliferative and apoptosis‐resistant phenotype of pulmonary artery smooth muscle cells (PASMCs) (Santos et al. 2023; Si et al. 2025). Mechanistically, poly (ADP‐ribose) polymerase 1 (PARP1) has emerged as a critical mediator in PAH and right ventricular failure, in part by amplifying NF‐κB–dependent inflammatory signaling and exacerbating mitochondrial injury (Shafiq et al. 2023; Shimauchi et al. 2022). Prior evidence further suggests that VD deficiency upregulates Hes1, a Notch pathway effector implicated in vascular remodeling (Jin et al. 2024), and Hes1 can interact with PARP1 to enhance its activation in a cell type‐dependent manner (Kannan et al. 2011), supporting a Hes1‐PARP1 axis as a plausible VD‐sensitive amplifier of NF‐κB signaling. In addition, TNFAIP3 (A20), a canonical negative regulator of NF‐κB (Zhang et al. 2025), has been reported as a VD‐responsive target gene (Gospodarska et al. 2025) and is implicated in restraining inflammation and preserving mitochondrial integrity (Zhou et al. 2025). Therefore, we hypothesized that VD supplementation, as a nutritional intervention, protects against pulmonary vascular remodeling in experimental PAH by downregulating the Hes1‐PARP1 axis while upregulating TNFAIP3, thereby suppressing aberrant NF‐κB activation, mitigating inflammation, and restoring mitochondrial homeostasis.

To validate this hypothesis, we systematically investigated the role of VD in experimental PAH models. We examined whether VD modulates inflammation and mitochondrial dysfunction by regulating the Hes1‐PARP1 axis and TNFAIP3, thereby restraining NF‐κB activation. By elucidating a dual regulatory framework through which VD influences NF‐κB signaling, this study aims to provide mechanistic insight into nutrition‐sensitive pathways relevant to CTD‐PAH‐associated vascular remodeling and to support the rationale for VD‐related nutritional strategies in early disease intervention.

## 2 Methods

### 2.1 Animal Model and Treatment

To investigate pulmonary vascular remodeling mechanisms relevant to CTD‐PAH, a monocrotaline (MCT)‐induced PAH rat model was employed. SD rats (6–8 weeks old, 180–220 g) were obtained from Bestcell Biotechnology Co. Ltd. (China) and housed in a SPF facility under standardized conditions (22°C–24°C, 50%–60% humidity, 12‐h light/dark cycle) with ad libitum access to chow and water. All animals were acclimatized for 1 week before experiments.

Rats were randomly assigned to three groups (*n* = 6): (1) Control group, receiving a subcutaneous injection of saline followed by daily gavage with olive oil; (2) MCT group, administered a single subcutaneous dose of monocrotaline (50 mg/kg; Sigma‐Aldrich, USA) with olive oil gavage (Shah et al. 2023); (3) MCT + VD group, administered MCT together with daily gavage of VD (100 IU dissolved in olive oil; Sinopharm, China) (Shah et al. 2023), initiated on the day of MCT injection and maintained for 4 weeks. No animals were excluded from the analysis, and no unexpected mortality occurred during the 4‐week experimental period. Approval for all animal procedures was obtained from the Ethics Committee of SuZhou WuZhong People's Hospital.

### 2.2 Hemodynamic Measurement

To quantify the severity of PAH in vivo and determine the hemodynamic impact of VD treatment, RVSP and mPAP were measured by invasive catheterization. Isoflurane (3%–5% for induction and 1.5%–2.5% for maintenance in oxygen) was used to anesthetize the rats, which were then placed on the surgical table. The right jugular vein was cannulated via a cervical approach using a heparinized PE‐50 catheter, which was advanced into the right ventricle and pulmonary artery. Hemodynamic signals were obtained by connecting the catheter to a pressure transducer and recording with the PowerLab acquisition system (ADInstruments, Australia), enabling continuous monitoring of right ventricular systolic pressure (RVSP) and mean pulmonary arterial pressure (mPAP).

### 2.3 Right Ventricular Hypertrophy Assessment

To assess right ventricular remodeling secondary to increased pulmonary vascular load, right ventricular hypertrophy was quantified by the RVHI index. Rats were euthanized and hearts were rapidly excised and rinsed in ice‐cold saline. The atria were excised, and the RV was isolated from the combined LV and septum (LV + S) by dissection along the septal insertion site. Each portion was blotted dry and weighed. The right ventricular hypertrophy index (RVHI) was expressed as:

$$\text{RVHI}=\frac{\mathrm{RV}}{\left(\mathrm{LV}+S\right)}$$

### 2.4 Histological Analysis of Pulmonary Vascular Remodeling

To directly evaluate pulmonary arterial wall thickening and lumen narrowing as structural readouts of vascular remodeling, lung sections were subjected to histomorphometric analysis. After perfusion with cold saline, lung samples were fixed in 4% paraformaldehyde (Sinopharm, China), processed into paraffin blocks, and sectioned at 4 μm. Hematoxylin–eosin (HE) staining was applied to assess arterial morphology. Randomly selected small pulmonary arteries (50–150 μm external diameter) were analyzed for wall thickness (WT%) and wall area (WA%) using the following formula:

$$\mathrm{WT}\%=\frac{\left(\mathrm{ED}-\mathrm{ID}\right)}{\mathrm{ED}}\times 100\%,\mathrm{WA}\%=\frac{\left(\mathrm{VA}-\mathrm{LA}\right)}{\mathrm{VA}}\times 100\%$$

where ED is external diameter, ID is internal diameter, VA is vessel area, and LA is lumen area. At least 10 vessels per rat were analyzed using ImageJ software by investigators blinded to group allocation.

### 2.5 Cell Culture and Treatments

To complement the in vivo remodeling results with mechanistic interrogation at the cellular level, PASMCs were used to model vascular cell dysfunction under PAH‐like stimuli. Rat PASMCs were obtained from iCell Bioscience (China) and grown in DMEM (Gibco, USA) containing 10% FBS, penicillin (100 U/mL), and streptomycin (100 μg/mL) at 37°C with 5% CO_2_. Cells at passages 3–6 were used.

To interrogate PASMC‐intrinsic inflammatory and mitochondrial mechanisms relevant to pulmonary vascular pathology in CTD‐PAH, a PAH‐like phenotype in PASMCs was induced by PDGF‐BB (15 ng/mL; PeproTech, USA) treatment for 48 h under hypoxia (2% O_2_) conditions. VD (calcitriol; Sigma‐Aldrich, USA) was dissolved in DMSO and adjusted to 50 nM (Shah et al. 2023), with vehicle controls included. Gain‐ and loss‐of‐function approaches were used to define the contribution of Hes1/PARP1 and TNFAIP3 to VD‐associated signaling changes. For gene manipulation, Hes1 or TNFAIP3 siRNAs or overexpression plasmids were transfected using Lipofectamine 3000 (Invitrogen, USA). In some experiments, cells were also exposed to the PARP1 inhibitor PJ34 (10 μM; Selleck, USA).

### 2.6 Immunofluorescence Staining

Immunofluorescence was performed to localize vascular markers and quantify cellular phenotypes. This assay was performed on 4‐μm paraffin lung sections and PASMCs grown on glass coverslips. Tissue sections underwent deparaffinization and antigen retrieval before permeabilization with 0.3% Triton X‐100 (Sinopharm). Both sample types were blocked with 5% BSA (Biofroxx, Germany) for 1 h at 25°C. Primary antibodies targeting α‐SMA, Ki67, p65, and four inflammatory cytokines were applied overnight at 4°C, followed by fluorescent secondary antibodies for 1 h protected from light. Nuclear counterstaining was performed using DAPI (Sigma‐Aldrich). For apoptosis detection, a TUNEL assay kit (Roche, Switzerland) was used together with α‐SMA immunostaining as per the manufacturer's instructions. Antifade‐mounted samples were observed using a fluorescence microscope (Olympus, Japan). Positive cell counts (Ki67^+^/α‐SMA^+^ or TUNEL^+^/α‐SMA^+^) and nuclear p65 translocation were analyzed using ImageJ software (NIH, USA) by investigators blinded to the experimental groups. For each sample, at least 3 randomly selected fields of view were analyzed under identical acquisition settings, and the mean value was used for statistical analysis. Detail information of antibodies was presented in Table S1.

### 2.7 Enzyme‐Linked Immunosorbent Assay (ELISA)

ELISA kits (ZCIBIO, China) were used to quantify IL‐6, TNF‐α, CCL2, and ICAM‐1 in rat serum, lung tissue, and culture supernatants following the manufacturer's protocol. Optical density was read at 450 nm with a microplate reader (RAYTO, China).

### 2.8 Mitochondrial Function Assays

To determine whether VD affects mitochondrial homeostasis under experimental conditions associated with pulmonary vascular remodeling, mitochondrial membrane potential, oxidative stress, and ATP production were assessed using established assays. Mitochondrial membrane potential was determined by JC‐1 staining, in which cells were incubated with the working solution (Beyotime, China) at 37°C for 20 min, followed by washing and fluorescence imaging. Red (aggregates)/green (monomers) fluorescence ratio was used as an index of membrane potential. Intracellular ROS levels were assessed with DHE or DCFH‐DA staining (Beyotime), and signal intensity was analyzed by fluorescence measurement. Intracellular ATP levels were determined with a luminescence ATP detection kit (Beyotime).

### 2.9 Western Blot

Proteins were extracted from lung tissues and PASMCs with RIPA buffer containing protease and phosphatase inhibitors (ASPEN, China). Equivalent protein loads were resolved by SDS‐PAGE, transferred onto PVDF membranes, and probed with antibodies targeting Hes1, PARP1, TNFAIP3, p‐p65, p65, p‐IκBα, IκBα, Bax, Bcl‐2, cleaved caspase‐3, Drp1, Mfn1, OPA1, and β‐actin. Following HRP‐linked secondary antibody incubation, bands were visualized with ECL (ASPEN) and quantified using ImageJ software. Detail information of antibodies was presented in Table S1.

### 2.10 Quantitative Real‐Time Polymerase Chain Reaction (qRT‐PCR)

RNA was isolated with TRIpure reagent (ELK Biotechnology, China) and reverse‐transcribed into cDNA using a commercial kit (ELK). Quantitative PCR was performed with SYBR Green Master Mix (ELK) on a Life Technologies real‐time PCR platform. Expression of Hes1, PARP1, and TNFAIP3 was normalized to Actin and analyzed by the $2^{-∆∆C_{t}}$ method. Primer sequences were presented in Table S2.

### 2.11 PARP1 Activity Assay

Because PARP1 enzymatic activity can modulate NF‐κB‐related transcriptional responses, PARP1 activity was measured to functionally link Hes1–PARP1 interaction to downstream signaling. A colorimetric assay kit for PARP1 (R&D Systems, USA) was employed following the supplier's instructions, with absorbance detected at 450 nm. Enzyme activity was calculated relative to control samples.

### 2.12 Co‐Immunoprecipitation (Co‐IP)

Co‐IP was performed to test whether Hes1 physically associates with PARP1 in PASMCs, providing molecular support for the proposed Hes1‐PARP1 regulatory axis. PASMCs were lysed in ice‐cold IP buffer containing protease inhibitors, and equal protein amounts were incubated overnight at 4°C with anti‐Hes1 or anti‐PARP1 antibodies. Protein A/G agarose beads (ThermoFisher, USA) were then added, and the resulting complexes were washed, eluted, and subjected to Western blotting.

### 2.13 Cell Counting Kit‐8 (CCK‐8) Assay

Proliferation of PASMCs was evaluated with a CCK‐8 assay (Beyotime). Cells were plated in 96‐well plates, subjected to the indicated treatments, and incubated with CCK‐8 solution for 2 h before reading absorbance at 450 nm.

### 2.14 Flow Cytometry for Apoptosis

Annexin V‐FITC/PI staining (Sungene, China) was applied to measure apoptosis. Cells were processed and analyzed by flow cytometer (Beckman, USA), and apoptotic percentages were determined.

### 2.15 Statistical Analysis

Data are expressed as mean ± standard deviation (SD). Statistical tests were carried out with GraphPad Prism 9.0. Data were assessed for suitability for parametric testing before analysis. Differences between two groups were analyzed by unpaired two‐tailed Student's *t*‐test, whereas comparisons among multiple groups were analyzed by one‐way ANOVA followed by Tukey's post hoc analysis. A *p*‐value < 0.05 was deemed significant. For in vivo experiments, *n* represents the number of animals analyzed per group. For in vitro experiments, *n* represents independent biological replicates.

## 3 Results

### 3.1 VD Improves Hemodynamics, Attenuates Ventricular Hypertrophy and Vascular Remodeling, and Modulates PASMC Function in PAH Rats

**FIGURE 1:**
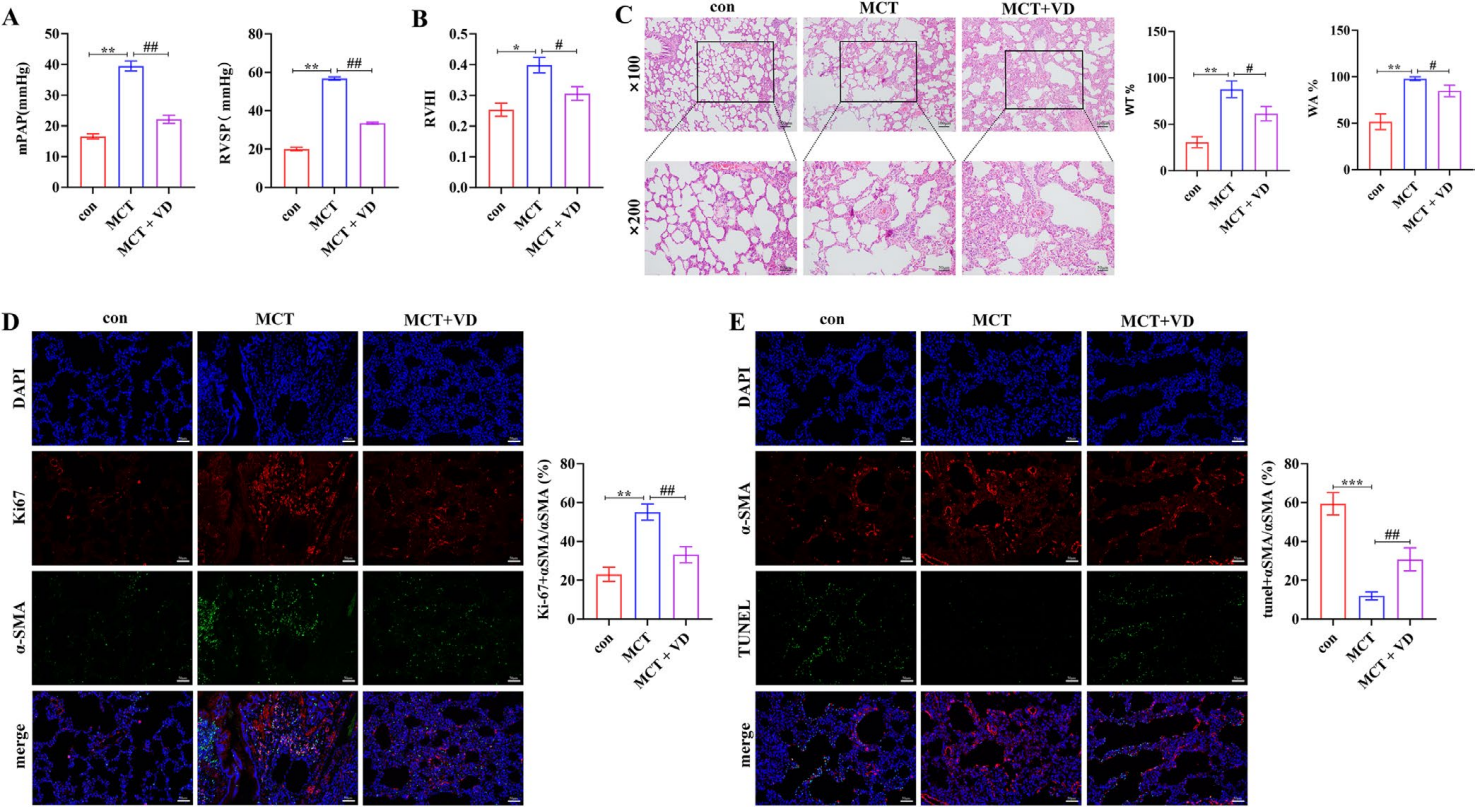
VD improves hemodynamics, attenuates right ventricular hypertrophy, and reduces pulmonary vascular remodeling in PAH rats. (A) Quantification of mean pulmonary arterial pressure (mPAP) and right ventricular systolic pressure (RVSP). (B) Right ventricular hypertrophy index (RVHI). (C) Representative HE staining of pulmonary arteries (scale bar = 100 μm for ×100; 50 μm for ×200) and quantification of wall thickness (WT%) and wall area (WA%). (D) Immunofluorescence staining of Ki67 and α‐SMA in pulmonary arteries and quantification of Ki67^+^α‐SMA^+^/α‐SMA^+^ PASMCs. (E) TUNEL and α‐SMA staining of PASMCs and quantification of TUNEL^+^α‐SMA^+^/α‐SMA^+^ cells. Scale bar = 50 μm. *n* = 6. *p < 0.05, **p < 0.01, ***p < 0.001; ^#^ *p* < 0.05, ^##^ *p* < 0.01.

As an initial step in examining VD supplementation as a nutritional intervention in PAH, hemodynamic function, right ventricular remodeling, and pulmonary vascular structure were evaluated in the rat model. MCT treatment markedly increased RVSP and mPAP, whereas VD significantly reduced both, indicating improved hemodynamic function (*p* < 0.05; Figure 1A). RVHI was also elevated in the MCT group, reflecting right ventricular remodeling, but was alleviated by VD treatment (*p* < 0.05; Figure 1B). Histological analysis with HE staining revealed increased WT% and WA% in small pulmonary arteries in the MCT group, which were substantially improved by VD (Figure 1C). Immunofluorescence staining further showed excessive PASMCs proliferation (Ki67+ and α‐SMA+ cells) following MCT, which was suppressed by VD (Figure 1D). TUNEL staining revealed reduced PASMC apoptosis in the MCT group, whereas VD restored apoptotic activity (Figure 1E). Collectively, these results indicate that VD supplementation mitigates key pathological features of experimental PAH including pulmonary hemodynamic impairment, right ventricular remodeling, and PASMC dysregulation.

### 3.2 VD Alleviates Inflammation and Improves Mitochondrial Function in PAH Rats

**FIGURE 2:**
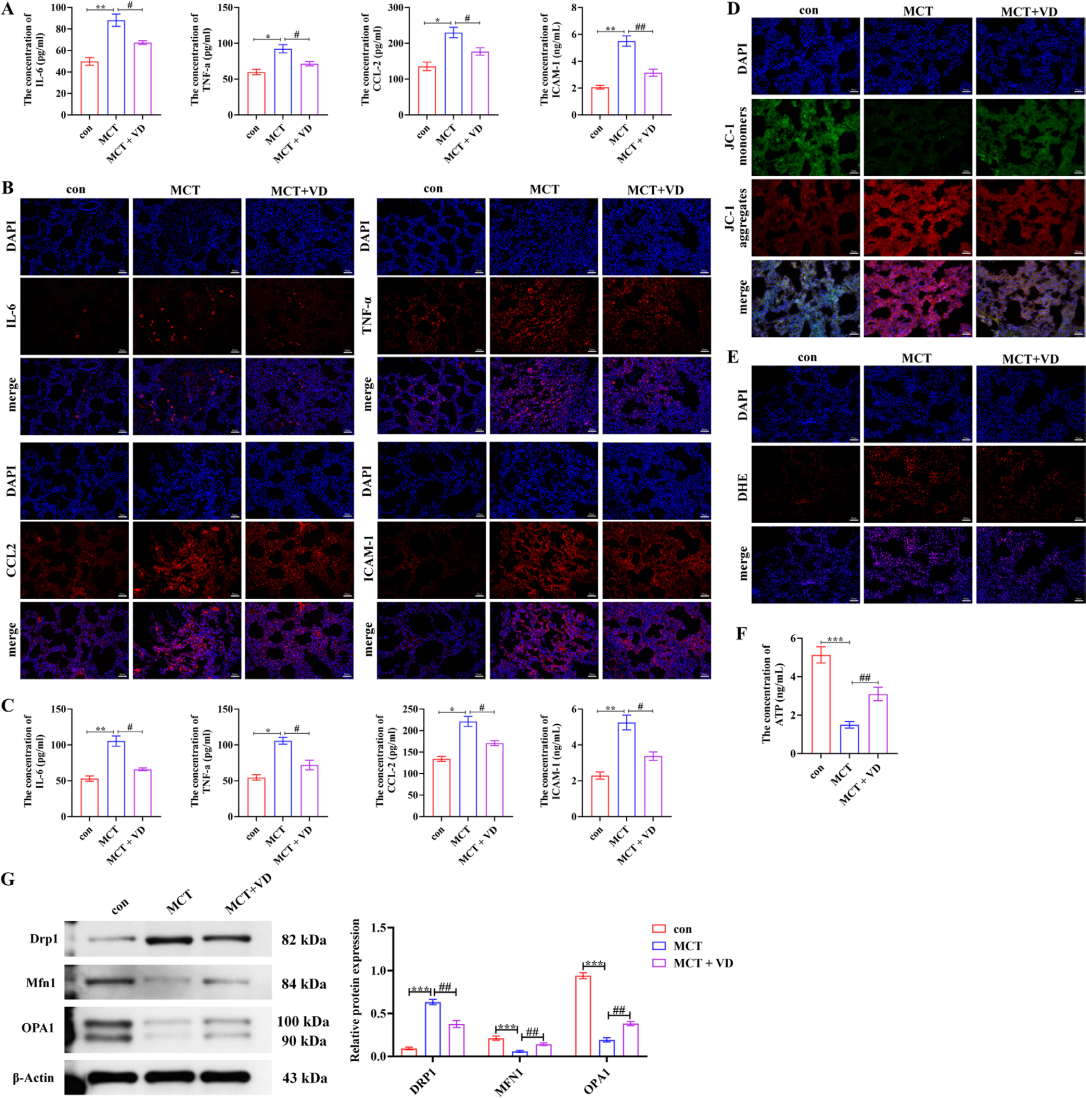
VD alleviates inflammation and restores mitochondrial homeostasis in PAH rats. (A–C) ELISA and immunofluorescence analysis of IL‐6, TNF‐α, CCL2, and ICAM‐1 in lung tissues and serum. (D) Mitochondrial membrane potential (JC‐1), (E) ROS production, and (F) ATP content. (G) Western blot of mitochondrial dynamics proteins (Drp1, Mfn1, OPA1) with quantification. Scale bar = 50 μm. *n* = 6. *p < 0.05, **p < 0.01, ***p < 0.001; ^#^ *p* < 0.05, ^##^ *p* < 0.01.

We next examined the effects of VD on inflammation and mitochondrial homeostasis in PAH rats. ELISA and immunofluorescence analyses showed that MCT markedly elevated IL‐6, TNF‐α, CCL2, and ICAM‐1 in lung tissue and serum, reflecting robust inflammatory activation; VD significantly reduced these levels (*p* < 0.05, *p* < 0.01; Figure 2A–C). MCT also induced mitochondrial dysfunction, characterized by membrane hyperpolarization, increased ROS, and reduced ATP, whereas VD ameliorated these abnormalities and partially normalized mitochondrial function (Figure 2D–F). At the molecular level, MCT upregulated the fission protein Drp1 while downregulating the fusion proteins Mfn1 and OPA1; these changes were reversed by VD treatment (*p* < 0.01; Figure 2G). These observations support inflammation and mitochondrial function as nutrition‐responsive processes that can be modulated by VD supplementation.

### 3.3 VD Inhibits NF‐κB Signaling in Lung Tissue by Modulating the Hes1–PARP1 Axis and TNFAIP3

**FIGURE 3:**
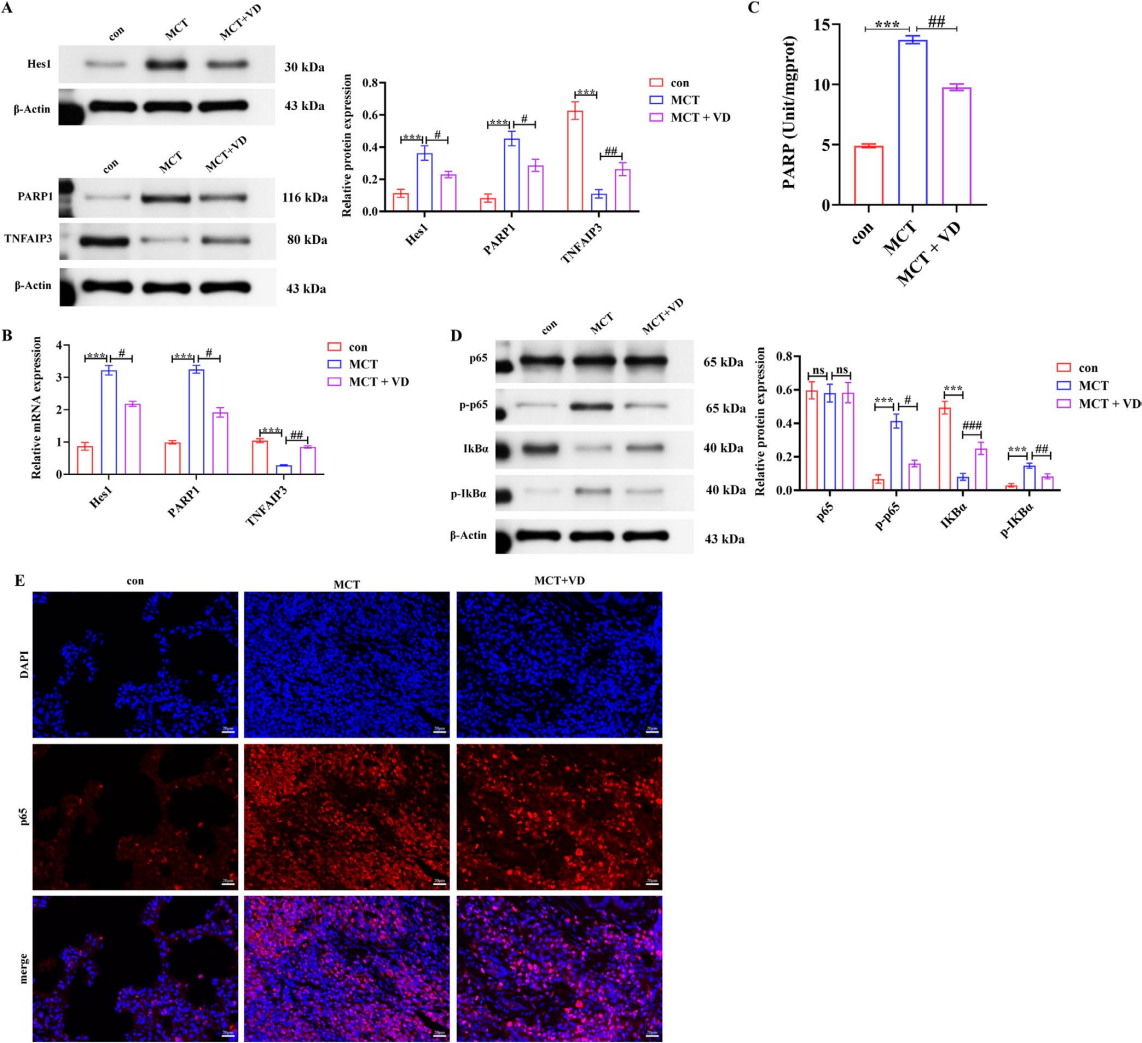
VD suppresses NF‐κB activation in lung tissue by modulating the Hes1–PARP1 axis and TNFAIP3. (A, B) Western blot and qRT‐PCR of Hes1, PARP1, and TNFAIP3 expression. (C) PARP1 enzymatic activity. (D, E) NF‐κB signaling assessed by p‐p65, p‐IκBα, IκBα expression, and p65 nuclear translocation. Scale bar = 50 μm. *n* = 6. *p* < 0.01, ***p < 0.001; ^#^ *p* < 0.05, ^##^ *p* < 0.01, ^###^ *p* < 0.001; ns, not significant.

To clarify the underlying mechanism, we examined the Hes1–PARP1 axis, TNFAIP3, and their impact on NF‐κB activation. MCT markedly upregulated Hes1 and PARP1 while downregulating TNFAIP3 at both protein and mRNA levels; VD partially reversed these changes (*p* < 0.05, *p* < 0.01; Figure 3A,B). PARP1 activity was elevated in the MCT group but suppressed by VD (*p* < 0.05; Figure 3C). Consistently, MCT increased p‐p65 and p‐IκBα, reduced IκBα, and promoted p65 nuclear translocation, whereas VD intervention inhibited the activation of NF‐κB (Figure 3D,E). Together, these results demonstrate that VD suppresses NF‐κB hyperactivation in lung tissue by downregulating the Hes1–PARP1 axis and upregulating TNFAIP3.

### 3.4 VD Suppresses NF‐κB Activation in PASMCs Through the Hes1–PARP1 Axis

**FIGURE 4:**
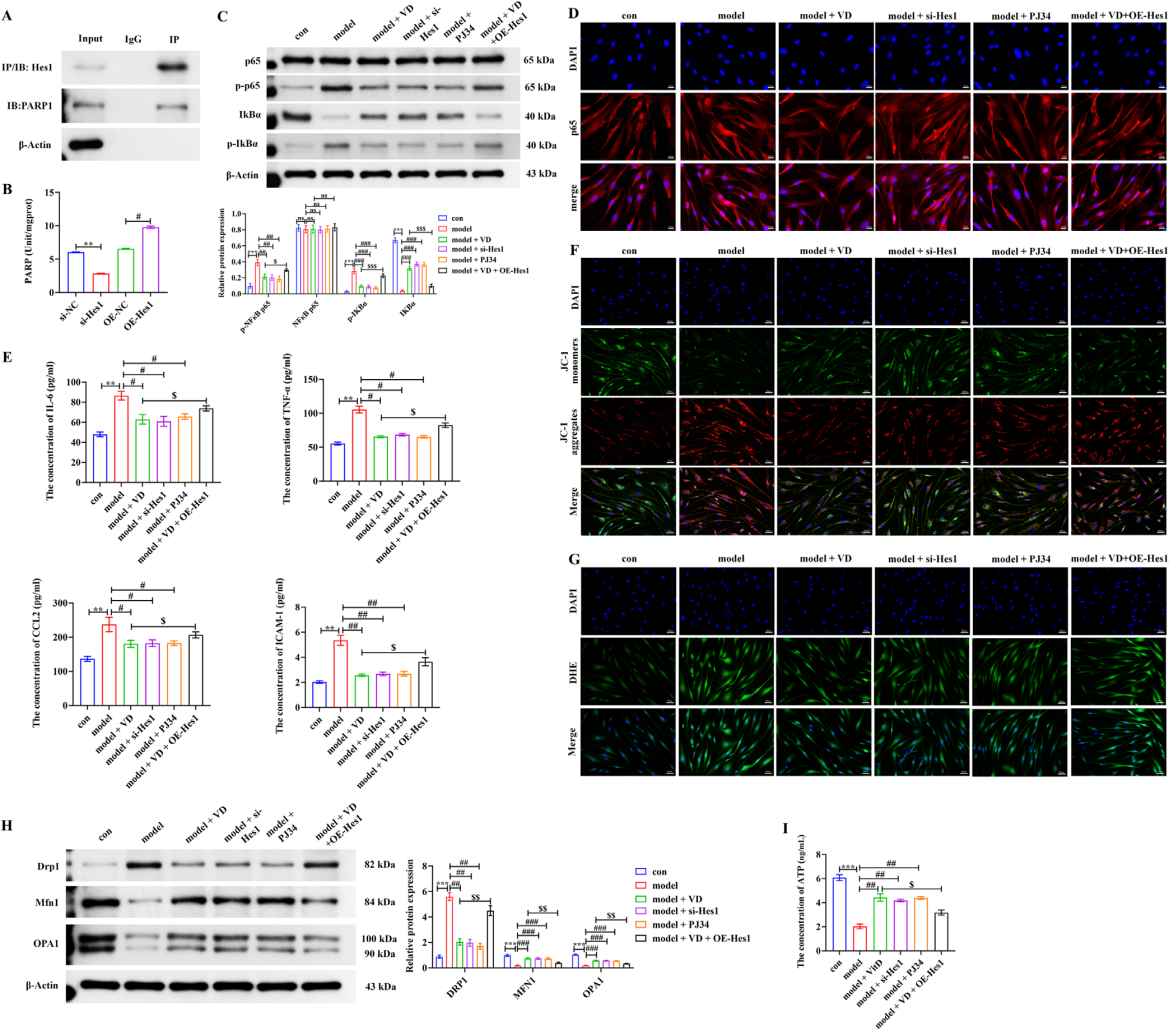
VD suppresses NF‐κB activation in PASMCs via the Hes1–PARP1 axis. (A) Co‐IP confirming Hes1–PARP1 interaction. (B) PARP1 activity in PASMCs with Hes1 overexpression or knockdown. (C, D) Western blot and immunofluorescence of NF‐κB signaling proteins. (E) ELISA of inflammatory cytokines. (F–I) Mitochondrial membrane potential, ROS, dynamics proteins, and ATP levels. Scale bar = 50 μm. Data are presented as mean ± SD from three independent biological replicates (*n* = 3). *p < 0.05, **p < 0.01, ***p < 0.001; ^#^ *p* < 0.05, ^##^ *p* < 0.01, ^###^ *p* < 0.001; ^$^ *p* < 0.05, ^$$^ *p* < 0.01, ^$$$^ *p* < 0.001; ns, not significant.

Based on the in vivo results, we further investigated the Hes1–PARP1 axis in PASMCs. PASMCs were stimulated with PDGF‐BB under hypoxia to establish the model, confirmed by excessive proliferation (Figure S1). Co‐IP assays demonstrated an interaction between Hes1 and PARP1 (Figure 4A). Functional study further showed that Hes1 overexpression enhanced PARP1 activity, whereas knockdown reduced it, with transfection efficiency validated by Western blot and qRT‐PCR (Figure 4B, Figure S2). Regarding NF‐κB signaling, treatment with VD, si‐Hes1, or the PARP1 inhibitor PJ34 reduced p‐p65 and p‐IκBα levels and blocked p65 nuclear translocation, whereas Hes1 overexpression partially reversed VD's effects (Figure 4C,D). Functionally, these interventions also reduced inflammatory cytokine secretion (*p* < 0.05, *p* < 0.01; Figure 4E) and ameliorated PDGF‐BB and hypoxia‐induced mitochondrial dysfunction, as evidenced by restored membrane potential (Figure 4F), reduced ROS (Figure 4G), normalized expression of mitochondrial dynamics proteins (Figure 4H), and increased ATP generation (Figure 4I). Notably, in the model + VD + OE‐Hes1 group, the beneficial effects of VD were partially reversed by Hes1 overexpression. The above results demonstrate that VD mitigates inflammation and mitochondrial dysfunction in PASMCs by modulating the Hes1–PARP1 axis to inhibit NF‐κB signaling.

### 3.5 VD Upregulates TNFAIP3 to Suppress NF‐κB Signaling in PASMCs

**FIGURE 5:**
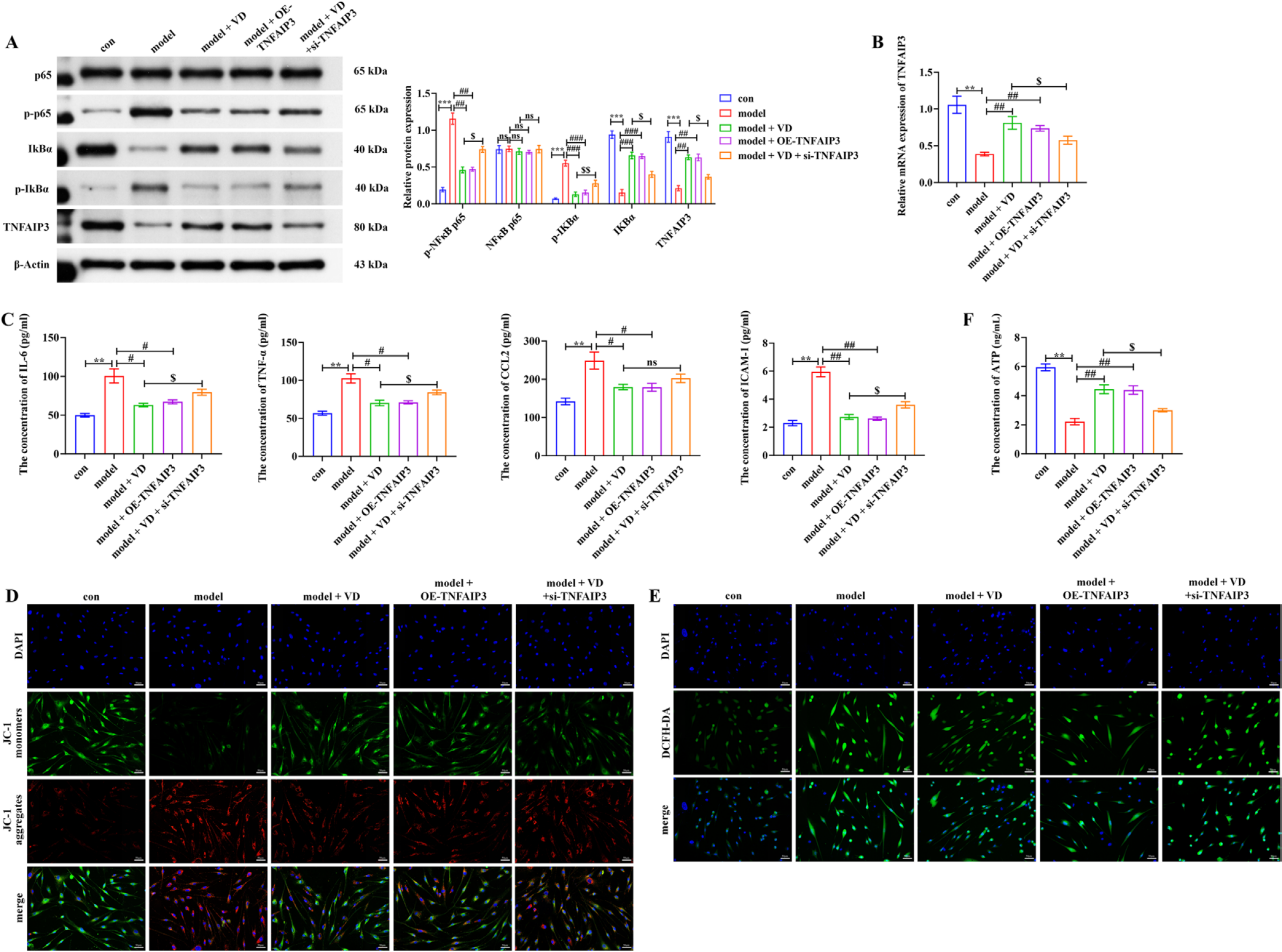
TNFAIP3 mediates the inhibitory effects of VD on NF‐κB signaling, inflammation, and mitochondrial dysfunction in PASMCs. (A) Western blot analysis of TNFAIP3 and NF‐κB signaling proteins. (B) Relative mRNA expression of TNFAIP3 was evaluated by qRT‐PCR (C) ELISA of inflammatory cytokines. (D–F) Mitochondrial membrane potential, ROS, and ATP production. Scale bar = 50 μm. Data are presented as mean ± SD from three independent biological replicates (*n* = 3). **p < 0.01, ***p < 0.001; ^#^ *p* < 0.05, ^##^ *p* < 0.01, ^###^ *p* < 0.001; ^$^ *p* < 0.05; ns, not significant.

Given the role of TNFAIP3 as a negative regulator of NF‐κB, we next examined its involvement in VD's effects. PDGF‐BB and hypoxia dramatically reduced TNFAIP3 expression, whereas VD supplementation or TNFAIP3 overexpression (OE‐TNFAIP3) restored its levels, accompanied by reduced p‐p65 and p‐IκBα and stabilization of IκBα. Conversely, TNFAIP3 knockdown under VD treatment (VD + si‐TNFAIP3) weakened these effects (Figure 5A,B), indicating that TNFAIP3 is involved in and mediates the protective effects of VD. ELISA further confirmed that VD or OE‐TNFAIP3 reduced inflammatory cytokine secretion (*p* < 0.05, *p* < 0.01), whereas inflammation rebounded in the VD + si‐TNFAIP3 group (*p* < 0.05) (Figure 5C). Moreover, both VD and OE‐TNFAIP3 alleviated MCT‐induced mitochondrial hyperpolarization and reduced ROS production, whereas these benefits were significantly diminished by TNFAIP3 knockdown under VD treatment (Figure 5D,E). Consistently, ATP assays showed that VD and OE‐TNFAIP3 restored ATP generation reduced by PDGF‐BB and hypoxia, but this effect was markedly blunted in the VD + si‐TNFAIP3 group (Figure 5F). These findings identify TNFAIP3 as a key downstream effector of VD, mediating NF‐κB inhibition, restraining inflammation, and preserving mitochondrial homeostasis.

### 3.6 VD Suppresses PASMCs Proliferation, Apoptosis Resistance, and Phenotypic Switching via the Hes1–PARP1 Axis

**FIGURE 6:**
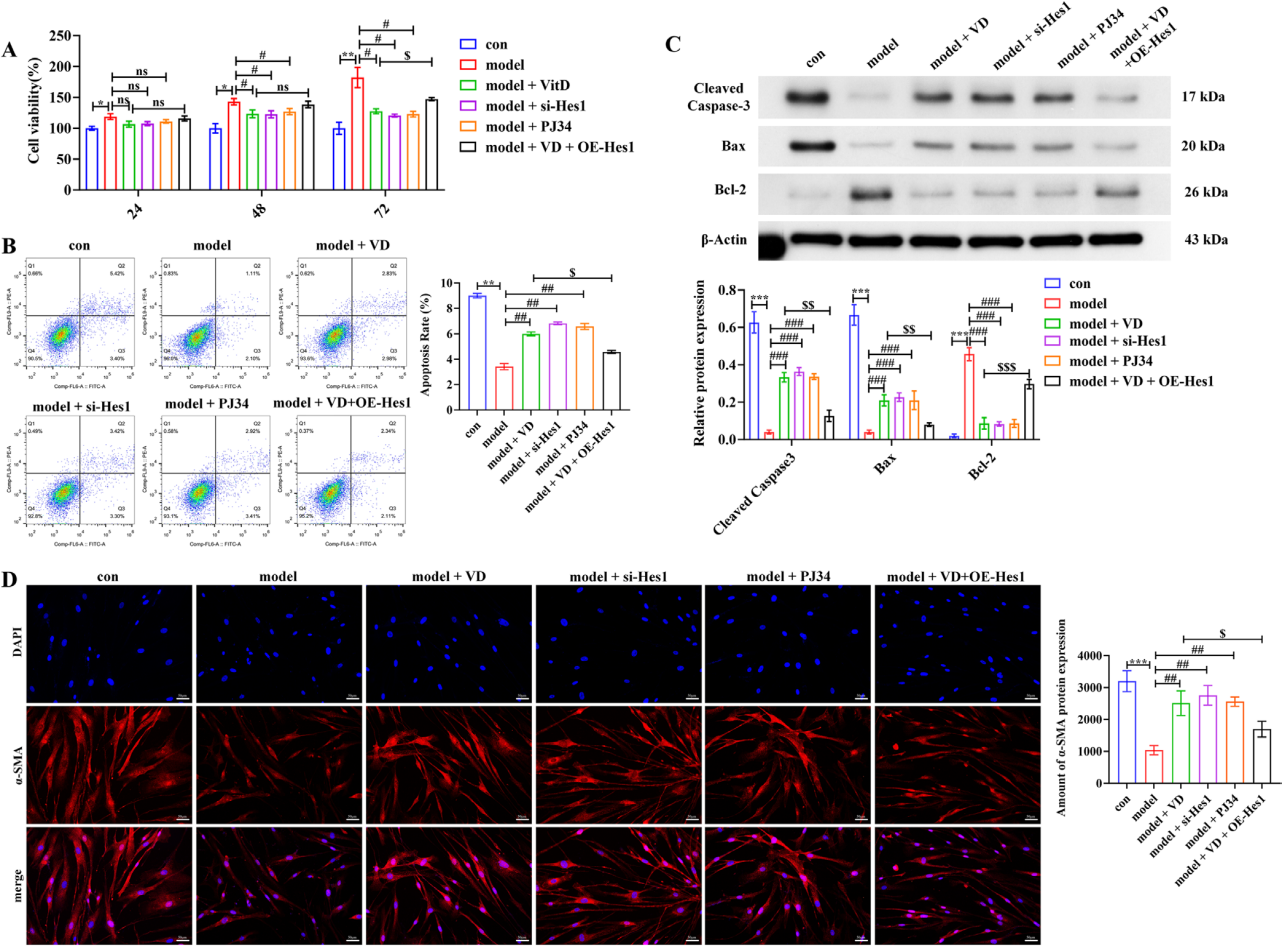
VD suppresses PASMCs proliferation, apoptosis resistance, and phenotypic switching via the Hes1–PARP1 axis. (A) CCK‐8 proliferation assay. (B) Flow cytometry analysis of apoptosis. (C) Western blot of apoptosis‐related proteins (Bax, cleaved caspase‐3, Bcl‐2). (D) Immunofluorescence staining of α‐SMA. Scale bar = 50 μm. Data are presented as mean ± SD from three independent biological replicates (*n* = 3). *p < 0.05, **p < 0.01, ***p < 0.001; ^#^ *p* < 0.05, ^##^ *p* < 0.01, ^###^ *p* < 0.001; ^$^ *p* < 0.05, ^$$^ *p* < 0.01, ^$$$^ *p* < 0.001; ns, non‐significant.

To further clarify the role of the Hes1–PARP1 axis in PASMCs dysfunction, we examined cell proliferation, apoptosis, and phenotypic switching. CCK‐8 assays showed that PDGF‐BB and hypoxia markedly enhanced PASMCs proliferation, whereas VD, si‐Hes1, or PJ34 significantly suppressed this excessive growth. Notably, Hes1 overexpression under VD treatment partially reversed the effect of VD at 72 h (*p* < 0.05; Figure 6A). Flow cytometry revealed reduced apoptosis in the MCT group, which was restored by VD, si‐Hes1, or PJ34 (*p* < 0.01), but attenuated by Hes1 overexpression in the presence of VD (*p* < 0.05) (Figure 6B). Western blot analysis confirmed these findings: VD, si‐Hes1, or PJ34 increased Bax and cleaved caspase‐3 expression while decreasing Bcl‐2 (*p* < 0.001), whereas Hes1 overexpression counteracted these changes (*p* < 0.01, *p* < 0.001) (Figure 6C). Regarding phenotypic modulation, immunofluorescence staining demonstrated that PDGF‐BB and hypoxia induced a contractile‐to‐synthetic phenotypic switch in PASMCs, marked by reduced α‐SMA expression. This transition was blocked by VD, si‐Hes1, or PJ34 (*p* < 0.01), but attenuated by Hes1 overexpression under VD treatment (*p* < 0.05) (Figure 6D). These results demonstrate that VD improves PDGF‐BB and hypoxia‐induced PASMCs dysfunction by modulating the Hes1–PARP1 axis to inhibit proliferation and phenotypic switching while restoring apoptosis.

### 3.7 VD Inhibits PASMCs Proliferation, Apoptosis Resistance, and Phenotypic Switching by Upregulating TNFAIP3

**FIGURE 7:**
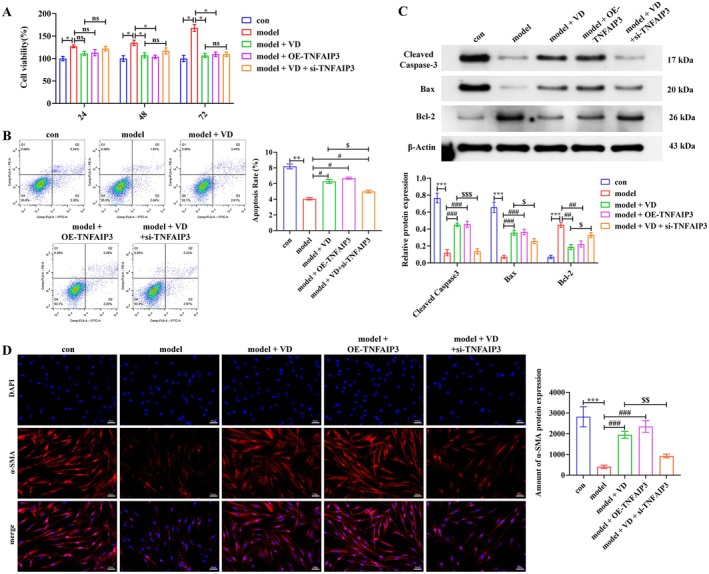
VD inhibits PASMCs dysfunction by upregulating TNFAIP3. (A) CCK‐8 proliferation assay. (B) Flow cytometry analysis of apoptosis. (C) Western blot of Bax, cleaved caspase‐3, and Bcl‐2. (D) Immunofluorescence staining of α‐SMA for phenotypic switching. Scale bar = 50 μm. Data are presented as mean ± SD from three independent biological replicates (*n* = 3). *p < 0.05, **p < 0.01, ***p < 0.001; ^#^ *p* < 0.05, ^##^ *p* < 0.01, ^###^ *p* < 0.001; ^$^ *p* < 0.05, ^$$^ *p* < 0.01, ^$$$^ *p* < 0.001; ns, not significant.

To further clarify the role of TNFAIP3 in PASMCs dysfunction, we established TNFAIP3 overexpression and knockdown models in combination with VD treatment. CCK‐8 assays showed that PDGF‐BB and hypoxia markedly increased PASMCs proliferation (*p* < 0.05), which was suppressed by VD or OE‐TNFAIP3 (*p* < 0.05) (Figure 7A). Flow cytometry revealed reduced apoptosis after MCT (*p* < 0.01), which was rescued by VD or OE‐TNFAIP3 (*p* < 0.05), but attenuated in the VD + si‐TNFAIP3 group (*p* < 0.05) (Figure 7B). Western blot analysis confirmed that VD or OE‐TNFAIP3 increased Bax and cleaved caspase‐3 while decreasing Bcl‐2 (*p* < 0.01, *p* < 0.001), whereas si‐TNFAIP3 reversed these changes (*p* < 0.05, *p* < 0.001) (Figure 7C). Regarding phenotypic modulation, immunofluorescence staining showed that PDGF‐BB and hypoxia markedly reduced α‐SMA expression (*p* < 0.001), indicating a shift toward the synthetic phenotype. This was prevented by VD or OE‐TNFAIP3 (*p* < 0.001) but weakened by VD + si‐TNFAIP3 (*p* < 0.01) (Figure 7D). Collectively, these results identify TNFAIP3 as a critical mediator of VD's protective effects in PASMCs, suppressing proliferation and apoptosis resistance while preventing phenotypic switching.

## 4 Discussion

In this study, we investigated VD as a modifiable micronutrient‐related factor in experimental PAH models. Across in vivo and in vitro readouts, VD improved hemodynamics, reduced right ventricular hypertrophy and vascular remodeling, and normalized PASMC proliferation, phenotype, and apoptosis. It also alleviated inflammation and mitochondrial dysfunction. Mechanistically, VD limited excessive NF‐κB signaling through coordinated downregulation of the Hes1‐PARP1 axis and upregulation of TNFAIP3.

The findings of this study align with clinical and epidemiologic observations linking low VD status to worse PAH phenotypes and suggesting potential benefit from repletion in deficient individuals (Callejo et al. 2021; Tanaka et al. 2017). Notably, beyond its beneficial effects on hemodynamics and right ventricular remodeling, VD also directly modulates PASMCs—the key effector cells that drive vascular remodeling and progression in PAH. Under hypoxic and PDGF‐BB stimulation, PASMCs exhibited excessive proliferation, resistance to apoptosis, and phenotypic switching, all of which were markedly reversed by VD treatment, suggesting that VD acts on a central step of vascular remodeling.

At the pathological level, we observed concurrent inflammation and mitochondrial abnormalities: IL‐6, TNF‐α, CCL2, and ICAM‐1 levels were elevated in both PAH rats and PASMCs, accompanied by sustained NF‐κB activation. Mitochondrial homeostasis was also disrupted, with membrane hyperpolarization, increased ROS, reduced ATP generation, and imbalanced dynamics. These findings are consistent with previous reports showing that elevated inflammatory cytokines and immune cell infiltration are observed in PAH, whereas mitochondrial dysfunction promotes metabolic abnormalities and apoptosis resistance in PASMCs, jointly driving vascular remodeling and right ventricular overload (Lockett et al. 2025; Zhao et al. 2024). Notably, our study revealed mitochondrial hyperpolarization, which is consistent with most reports (Courboulin et al. 2011; Tuineau et al. 2023); however, some studies have also described depolarization of the mitochondrial membrane potential in PASMCs under PAH conditions (Dong et al. 2014; Sevilla‐Montero et al. 2022), which may reflect heterogeneity in mitochondrial electrophysiology across disease stages or models. Furthermore, VD treatment reduced inflammatory cytokine levels, suppressed NF‐κB activation, and ameliorated mitochondrial dysfunction. These results align with the anti‐inflammatory and mitochondrial‐protective effects of VD reported in chronic intestinal and lung diseases (Chen et al. 2022; Yu et al. 2021) and, importantly, provide the first systematic validation of these effects in a PAH model, supporting the nutritional relevance of VD during early pulmonary vascular remodeling.

At the molecular level, this study demonstrates that VD attenuates key PAH‐related pathological processes by regulating NF‐κB signaling through two coordinated and complementary mechanisms. First, we found that Hes1, a Notch pathway effector involved in cell differentiation, proliferation, and inflammation, interacts with PARP1 in PASMCs and enhances its activity; this observation is consistent with the findings of Kannan et al. (2011). Since PARP1 serves as a transcriptional coactivator of NF‐κB, whose overactivation amplifies inflammation and aggravates mitochondrial injury (Jin et al. 2025), our results indicate that VD suppresses NF‐κB hyperactivation by modulating the Hes1–PARP1 axis, thereby conferring both anti‐inflammatory and mitochondrial‐protective effects. Second, VD markedly upregulated TNFAIP3, a classical negative regulator of NF‐κB known to protect against immune‐mediated diseases. TNFAIP3 expression was reduced in PAH models but restored by VD supplementation or TNFAIP3 overexpression, which effectively inhibited NF‐κB activation; conversely, TNFAIP3 knockdown attenuated VD's protective effects. These findings identify TNFAIP3 as an essential downstream effector of VD and, together with prior evidence that it is a direct target of VD (Jaroslawska et al. 2024), provide the first systematic validation of this mechanism in the current PAH model system, with relevance to CTD‐PAH. Collectively, our study highlights a dual regulatory mechanism by which VD modulates NF‐κB signaling: it suppresses upstream activation through the Hes1–PARP1 axis and concurrently strengthening endogenous inhibitory control via TNFAIP3, with both regulatory effects converging on NF‐κB to ensure effective restraint of excessive NF‐κB activation.

From a nutrition science perspective, these findings suggest that vitamin D status, rather than a pathway‐specific pharmacological approach, may influence inflammatory and mitochondrial homeostasis during early pulmonary vascular remodeling. Given the frequent occurrence of VD deficiency in PAH (Callejo et al. 2020), our data provide mechanistic evidence linking reduced VD signaling to enhanced NF‐κB‐related inflammation and mitochondrial dysfunction, potentially via coordinated regulation of the Hes1‐PARP1 axis and TNFAIP3‐mediated negative feedback. Collectively, these results indicate that vitamin D status is closely associated with inflammatory and mitochondrial homeostasis during early pulmonary vascular remodeling. Future nutrition‐focused studies may therefore consider 25(OH)D status and vitamin D adequacy, whereas food‐based approaches to improve vitamin D intake (e.g., dietary fortification) require further investigation beyond the present experimental models.

This study has several limitations. First, the MCT‐induced rat model and in vitro PASMCs used in this study reflect pulmonary vascular remodeling but not the systemic autoimmune features of CTD‐PAH; thus, our findings are limited to PASMC‐intrinsic mechanisms and require validation in CTD‐PAH patient samples or autoimmune models. Second, we did not directly quantify nutritional status biomarkers (e.g., serum 25(OH)D) or related mineral metabolism indices (e.g., calcium, phosphate, PTH), limiting inference about the magnitude of status change needed to achieve the observed effects. In addition, although active vitamin D metabolites (e.g., calcitriol) were used as mechanistic tools, such exposure does not directly reflect dietary VD intake; therefore, translational interpretation should be anchored to nutritional biomarkers, particularly circulating 25(OH)D, and to deficiency‐correction paradigms. Finally, the optimal dosage and long‐term effects of VD supplementation have not yet been systematically evaluated in clinical studies, underscoring the need for large‐scale prospective trials to establish its safety and efficacy.

In summary, this study demonstrates that VD attenuates key pathological features in experimental PAH models by simultaneously downregulating the Hes1–PARP1 axis and upregulating TNFAIP3, thereby constraining excessive NF‐κB activation and coordinately regulating inflammation and mitochondrial homeostasis. These findings provide mechanistic insight into how vitamin D modulates inflammatory and mitochondrial homeostasis in pulmonary vascular pathology relevant to CTD‐PAH‐associated remodeling, thereby offering a nutrition‐oriented rationale grounded in defined molecular pathways.

## Author Contributions

X.L. and Y.J. conceived the study and designed the experiments. Y.J. completed the experiment, analyzed the data and wrote the manuscript. C.L., Y.L., and J.C. analyzed the data. C.L., X.L., and Y.J. discussed the results and revised the manuscript.

## Funding

This work was financially supported by the Suzhou Gusu Health Talent Project (no: GSWS2022113) and Wuzhong District Dongwu Health Talent Program (no. DWWS2025004).

## Ethics Statement

The study protocol was approved by the Ethics Committee of SuZhou WuZhong People's Hospital (no. 2025KY0024).

## Conflicts of Interest

The authors declare no conflicts of interest.

## Supporting information


**Figure S1:** fsn371620‐sup‐0001‐FigureS1.tif.

**Figure S2:** fsn371620‐sup‐0002‐FigureS2.tif.

**Table S1:** fsn371620‐sup‐0003‐TablesS1‐S2.docx.
**Table S2:** fsn371620‐sup‐0003‐TablesS1‐S2.docx.

## Data Availability

The data that support the findings of this study are available from the corresponding author upon reasonable request.
