# Supplementary figures and images for: Vitamin D Attenuates Inflammation and Mitochondrial Dysfunction in Experimental Models Relevant to Connective Tissue Disease–Associated Pulmonary Arterial Hypertension
Source: Food Sci Nutr. 2026 Mar 12;14(3):e71620. doi: 10.1002/fsn3.71620 (PMC13093515; doi:10.1002/fsn3.71620)

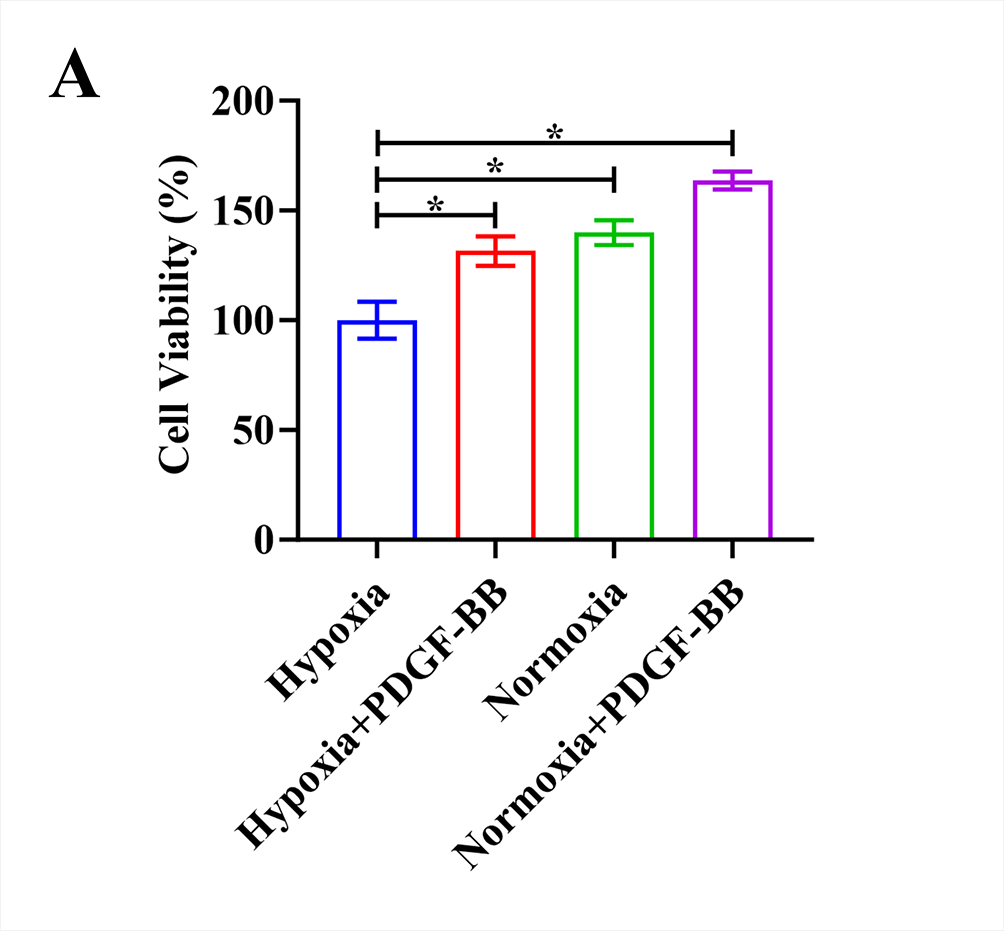

Supplement: Supplementary file 1 — Figure S1: fsn371620‐sup‐0001‐FigureS1.tif. [file FSN3-14-e71620-s002.tif]

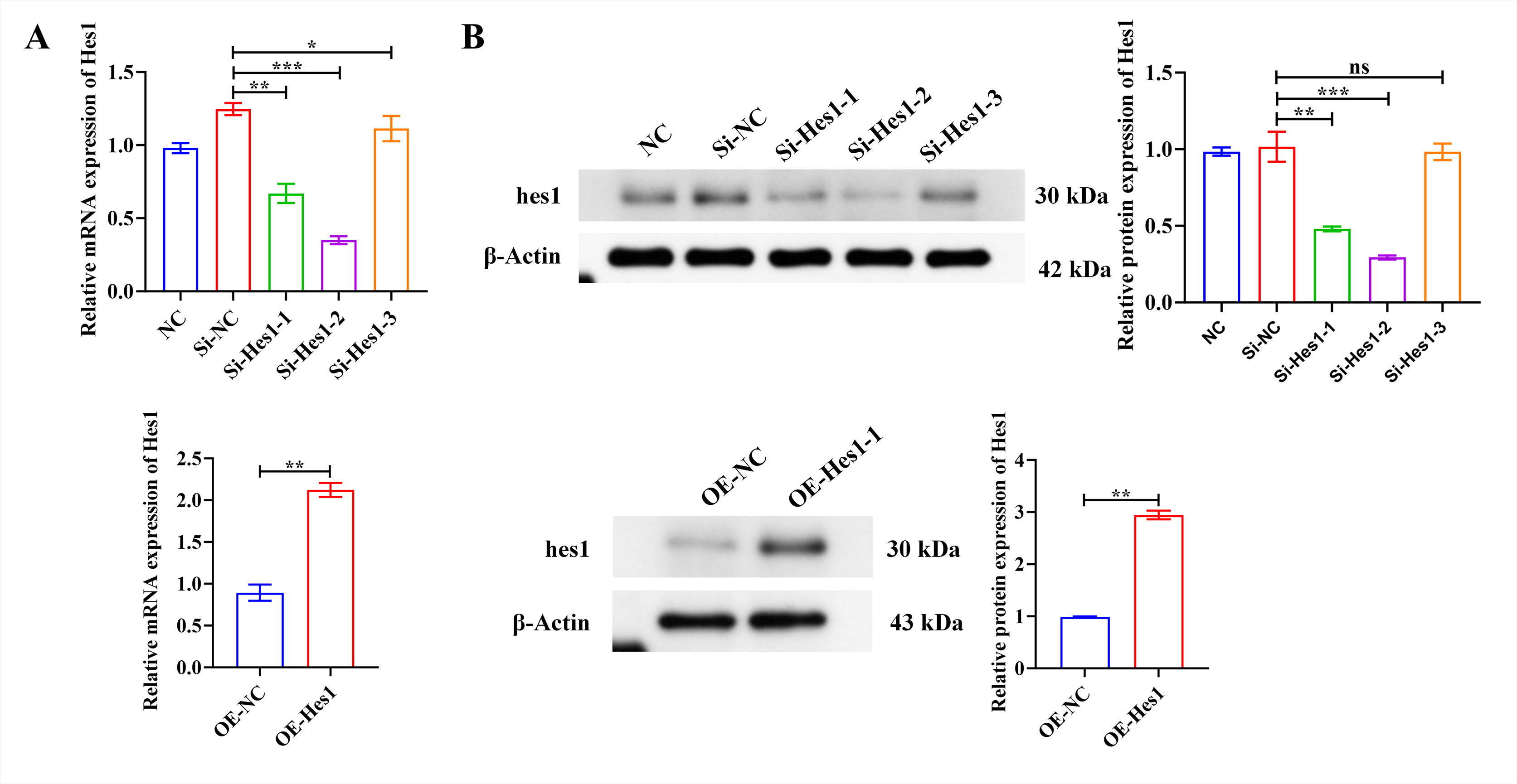

Supplement: Supplementary file 2 — Figure S2: fsn371620‐sup‐0002‐FigureS2.tif. [file FSN3-14-e71620-s001.tif]
