# Supplementary material for: Vitamin D Attenuates Inflammation and Mitochondrial Dysfunction in Experimental Models Relevant to Connective Tissue Disease–Associated Pulmonary Arterial Hypertension
Source: Food Sci Nutr. 2026 Mar 12;14(3):e71620. doi: 10.1002/fsn3.71620 (PMC13093515; doi:10.1002/fsn3.71620)
Supplement: Supplementary file 3 — Table S1: fsn371620‐sup‐0003‐TablesS1‐S2.docx. Table S2: fsn371620‐sup‐0003‐TablesS1‐S2.docx. [file FSN3-14-e71620-s003.docx]

**Supplementary Table 1. Antibodies used in this study**

| **Target protein** | **Application** | **Host species** | **Dilution ratio** | **Catalog number** | **Manufacturer** |
| --- | --- | --- | --- | --- | --- |
| Hes1 | WB, IF | Rabbit | 1:1000 (WB), 1:200 (IF) | Cell Signaling Technology (CST) | #11988 |
| PARP1 | WB, IF, Co-IP | Rabbit | 1:1000 (WB), 1:200 (IF) | Abcam | ab32138 |
| TNFAIP3 (A20) | WB | Rabbit | 1:1000 | Abcam | ab92324 |
| p-p65 (Ser536) | WB, IF | Rabbit | 1:1000 (WB), 1:200 (IF) | CST | #3033 |
| p65 (RelA) | WB, IF | Rabbit | 1:1000 (WB), 1:200 (IF) | CST | #8242 |
| p-IκBα (Ser32) | WB | Rabbit | 1:1000 | CST | #2859 |
| IκBα | WB | Rabbit | 1:1000 | CST | #4814 |
| Bax | WB | Rabbit | 1:1000 | CST | #2772 |
| Bcl-2 | WB | Rabbit | 1:1000 | CST | #3498 |
| Cleaved Caspase-3 | WB | Rabbit | 1:1000 | CST | #9661 |
| Drp1 | WB | Rabbit | 1:1000 | Abcam | ab184247 |
| Mfn1 | WB | Rabbit | 1:1000 | Abcam | ab57602 |
| OPA1 | WB | Rabbit | 1:1000 | Abcam | ab157457 |
| α-SMA | IF | Mouse | 1:200 | Sigma-Aldrich | A5228 |
| Ki67 | IF | Rabbit | 1:200 | Abcam | ab16667 |
| IL-6 | IF | Rabbit | 1:300 | Servicebio | GB11117 |
| TNFα | IF | Rabbit | 1:150 | Boster | BA0131 |
| CCL2 | IF | Rabbit | 1:50 | ABclonal | A7277 |
| ICAM-1 | IF | Rabbit | 1:150 | Proteintech Group | 16174-1-AP |
| Secondary antibody (Alexa Fluor 488, 594) | IF | Goat anti-rabbit/mouse | 1:500 | Invitrogen | A-11008 / A-11005 |
| β-actin | WB (loading control) | Mouse | 1:5000 | Proteintech | 66009-1-Ig |

**Supplementary Table 2. Primer sequences used for RT-qPCR**

| **Gene** | **RefSeq accession** | **Sequences (5’- 3’)** | |
| --- | --- | --- | --- |
| Hes1 | NM_024360.3 | sense | CGGACAAACCAAAGACAGCC |
|  |  | antisense | TCCGGAGGTGCTTCACTGTC |
| PARP1 | NM_013063 | sense | TGGACATCGAGGTTGCCTATAG |
|  |  | antisense | CACATACTTCCTGATGACCTCAGC |
| TNFAIP3 | NM_001427106 | sense | GAGCAATATGAGGAAAGCGGT |
|  |  | antisense | CACGTTCTGAACATCTCCAGTGT |
| Actin | NM_031144.3 | sense | CGTTGACATCCGTAAAGACCTC |
|  |  | antisense | TAGGAGCCAGGGCAGTAATCT |
